# Chemical Composition and Antioxidant Activity of *Tánara Ótó* (*Dracocephalum palmatum* Stephan), a Medicinal Plant Used by the North-Yakutian Nomads

**DOI:** 10.3390/molecules181114105

**Published:** 2013-11-14

**Authors:** Daniil N. Olennikov, Nadezhda K. Chirikova, Zhanna M. Okhlopkova, Ismayl S. Zulfugarov

**Affiliations:** 1 Institute of General and Experimental Biology, Siberian Division, Russian Academy of Science, Sakh’yanovoy str., 6, Ulan-Ude 670047, Russia; 2 Department of Biochemistry and Biotechnology, North-Eastern Federal University, 58 Belinsky Str., Yakutsk 677-027, Russia; E-Mail: hofnung@mail.ru; 3 Department of Biology, North-Eastern Federal University, 58 Belinsky Str., Yakutsk 677-027, Russia; E-Mails: biotechnologyYSU@rambler.ru (Z.M.O.); iszulfugarov@pusan.ac.kr (I.S.Z.); 4 Department of Molecular Biology, Pusan National University, Pusan 609-735, Korea; 5 Institute of Botany, Azerbaijan National Academy of Sciences, Baku AZ 1073, Azerbaijan

**Keywords:** *Dracocephalum palmatum* Stephan, Lamiaceae, North-Yakutian nomads, flavonoids, phenylpropanoids, antioxidant activity

## Abstract

*Dracocephalum palmatum* Stephan (Lamiaceae) is a medicinal plant used by the North-Yakutian nomads. From the crude ethanolic extract of the aerial parts of this plant, 23 compounds (phenylpropanoids, coumarins, flavonoids, and triterpenes) were isolated. Among these, eight compounds (salvianolic acid B, caftaric acid, cichoric acid, umbelliferone, aesculetin, apigenin-7-*O-*β-d-glucuronopyranoside, isorhoifolin, and luteolin-4'-*O-*β-d-glucopyranoside) were detected for the first time in the genus *Dracocephalum*. Their structures were elucidated based on chemical and spectral data. The levels of most of the compounds detected in the cultivated sample were close to that of the wild sample, indicating the reproducibility of the biologically active compounds of *D. palmatum* through cultivation. Investigation into the biological activity of *D. palmatum* under *in vitro* conditions demonstrated that its extracts have a strong antioxidant effect due to the presence of high concentrations of phenolic compounds.

## 1. Introduction

Yakutia (Sakha), a federal subject of Russia spanning 3,083,523 km^2^, is the largest subnational governing body by area in the World. If the federal subjects of Russia were compared with other countries, it would the eighth largest territory in the World. More than 40% of the territory is lies within the Arctic Circle. Due to its geographical location Yakutia is characterised by a variety of habitats and natural resources. Despite the uniqueness and diversity of the Yakutian flora, the scale of bioprospecting has been extremely low. As a part of the scientific programme on the investigation of plant diversity, we are now carrying out comprehensive studies of the chemical composition of Yakutian plants.

**Figure 1:**
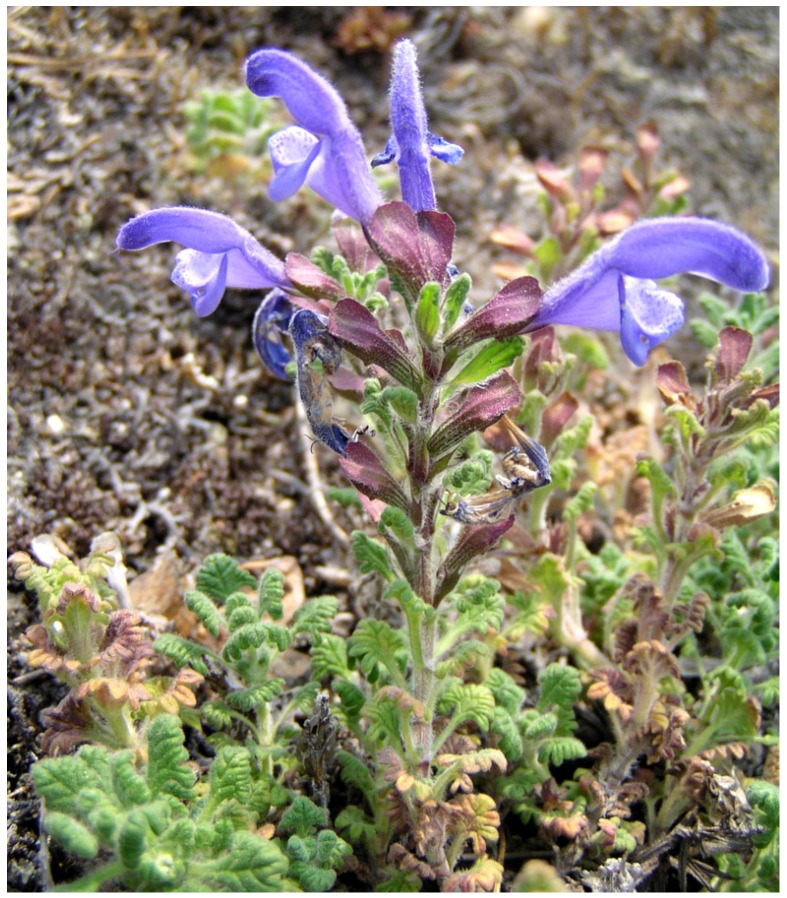
Palmate dragonhead—*Dracocephalum palmatum* Stephan.

Palmate dragonhead *Dracocephalum palmatum* Stephan (*D. schelechowii* Turcz. ex Ledeb., *Ruyschiana palmata* (Stephan ex Willd.) House; Subsection Keimodracontes Briq., Section Buguldea Benth., Subgenus Eudracocephalum Briq., Genus Dracocephalum L., Family Lamiaceae), is a perennial rhizomatous plant with numerous stems and ovate-rounded, pinnatifid leaves, and purple flowers on short stalks gathered in false whorls at the end of the stems in an oblong inflorescence (Figure 1). It grows on cliffs and sandy deposits, on gravelly and rocky slopes. It is endemic to the Arctic tundra (Chukotka, Anadyr), East Siberia (Yakutia), and the Russian Far East region [1].

The Nomads of North Yakutia call this plant *tánara ótó* (*maңapa omo*, the herb devoted to the supreme god *Tánara*). Young shoots and flowers have ethnobotanical uses as diuretic and chloretic remedy, for fumigation, treatment of gastro-intestinal tract disorders and alcoholism [2]. There are no scientific data about the chemical components and biological activity of *D. palmatum* herb.

*Dracocephalum* is a large genus of Lamiaceae family that includes about 60 species as perennial herbs (rarely semishrubs), growing in the territory of the extra-tropical Asia, Europe, and Russia. Ethnopharmacological information about species of this genus allows describing some of them as well-known and valuable sources of drugs. Among them, the most prominent species is *D. moldavica* L., traditionally used in the ethnomedicine of European countries for the treatment of hypertension and heart disease [3]. The aerial part of *D. heterophyllum* Benth. was used in Xinjiang (China) for treatment of asthma and gastropathy [4]. The best drug for diseases of the stomach and liver in Tibetian medicine was the herb of *D. nutans* [5]. In Buryatia (Russia) decoctions from *D. ryushiana* L. and *D. argunense* Fisch. ex Link. were recommended by ancient doctors (lamas) as choleretic remedies [6]. Despite a long history of human use of *Dracocephalum* species, scientific data about the chemical composition are known just for 16 species. Approximately a hundred compounds were isolated and identified as derivatives of terpenoids, flavonoids, alkaloids, and others [7]. These facts demonstrated the necessity of expanding scientific information about the species of the genus *Dracocephalum*. In this study, we present the results of a phytochemical investigation of *D. palmatum* herb of both wild (collected from Yakutia) and cultivated varieties and the antioxidant activity data of the ethanolic extracts from *D. palmatum* determined by *in vitro* methods.

## 2. Results and Discussion

### 2.1. Chemical Composition of *D. palmatum* Herb

**Figure 2:**
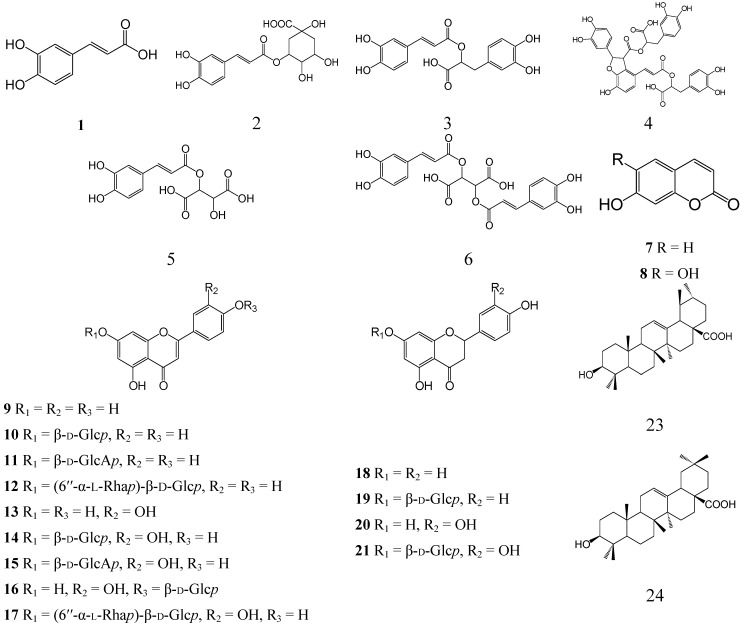
Chemical structures of compounds **1**–**23** isolated from *D. palmatum* herb.

The 60% EtOH extract of *D. palmatum* herb was partitioned with CHCl_3_, EtOAc, and *n*-BuOH to yield three fractions, which were separated by column chromatography (gel permeation, normal phase (NP-SiO_2_) and reversed phase silica gel (RP-SiO_2_), XAD, polyamide chromatography), preparative (prep.) high performance liquid chromatography (HPLC) and prep. thin layer chromatography (TLC)). Twenty three biologically active compounds, including six phenylpropanoids (caffeic acid (**1**), 3-*O*-caffeoylquinic acid (**2**), rosmarinic acid (**3**), salvianolic acid B (**4**), caftaric acid (**5**), cichoric acid (**6**)), two coumarins (umbelliferone (**7**), aesculetin (**8**)), thirteen flavonoids (apigenin (**9**), cosmosiin (**10**), apigenin-7-*O-*β-d-glucuronopyranoside (**11**), isorhoifolin (**12**), luteolin (**13**), cynaroside (**14**), luteolin-7-*O-*β-d-glucuronopyranoside (**15**), luteolin-4'-*O-*β-d-glucopyranoside (**16**), scolymoside (**17**), naringenin (**18**), naringenin-7-*O-*β-d-glucopyranoside (**19**), eriodictyol (**20**), eriodictyol-7-*O-*β-d-glucopyranoside (**21**)) and two triterpenes (ursolic acid (**22**), oleanolic acid (**23**)), were identified by comparing their optical rotations, UV, MS and NMR data with that reported in the literature (Figure 2).

It should be noted that compounds **1**–**3**, **9**, **10**, **13**, **14**, **22** and **23** are widely distributed in the Lamiaceae family and genus *Dracocephalum* [7]; compound **15** was previously isolated from *D. integrifolium* Bunge [8], **17** from *D. peregrinum* L. [9], **18**–**21** from *D. peregrinum*, *D. rupestre* Hance, and *D. tanguticum* Maxim. [7]. Eight components (compounds **4**–**8**, **11**, **12**, **16**) were detected for the first time in the genus *Dracocephalum*.

Despite insufficient chemical information of the phenolic compounds of *Dracocephalum* genus, available data suggest that flavonoid compounds, particularly derivatives of apigenin and luteolin, *i.e.*, flavones with 5,7,4' and 5,7,3',4' types of substitution are specific to this genus. These substances were found in the majority of *Dracocephalum* species studied, thus reflecting their important chemosystematic character. The systematic function of rosmarinic acid, a specific marker for Lamiaceae family, is not clear because its presence has not been detected in all *Dracocephalum* species. However, it should be noted that caffeic acid and its derivatives are an essential part of the *Dracocephalum* genus extracts.

β-d-Glcp—β-d-glucopyranose; β-d-GlcAp—β-d-glucuronopyranose; α-l-Rhap—α-l-rhamnopyranose.

### 2.2. HPLC-UV Analysis of the Main Phenolic Compounds in *D. palmatum*

**Figure 3:**
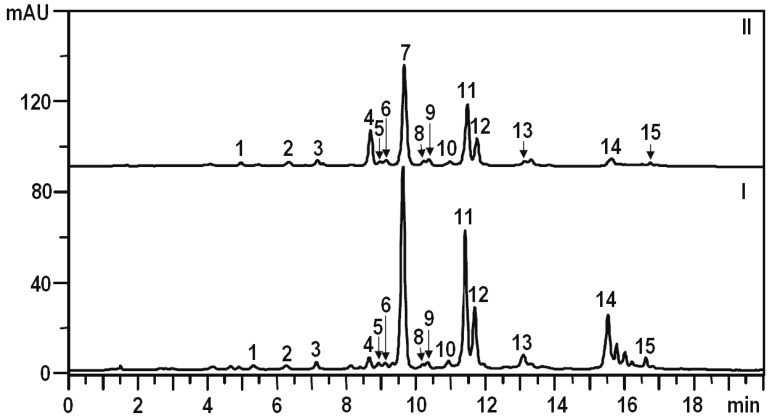
HPLC chromatograms of ethanolic extracts from wild and cultivated samples of *D. palmatum* at 270 nm. Samples: I—wild plant; II—cultivated plant.

A quantitative analysis of the phenolic compounds found in *D. palmatum* was performed using microcolumn HPLC with ultraviolet detection (HPLC-UV), which allowed separation of 15 dominant components (Figure 3). The predominant phenolic compounds from the wild sample of *D. palmatum* are flavonoids (20.987 mg/g) and the main group of the flavonoids is flavones (20.844 mg/g), with cynaroside (12.075 mg/g) and cosmosiin (5.683 mg/g) as dominating compounds (Table 1). Luteolin and its derivatives accounted for about 70% of the total flavonoids and apigenin and its derivatives accounted for less than 30%. The amount of flavanones did not exceed 1% of the total flavonoids. The content of glycosides was 11 times more than that of aglycones. Among glycosides, monoglycosides were dominant (19.420 mg/g) and the biosides (rutinosides) represented about 3% of the total amount of flavonoids. The content of phenylpropanoids in *D. palmatum* did not exceed 15% of the identified phenolic compounds. The dominant components of this group of metabolites were rosmarinic acid (1.614 mg/g) and salvianolic acid B (1.456 mg/g).

1. 3-*O*-Caffeoylquinic acid; 2. caffeic acid; 3. cichoric acid; 4. scolymoside; 5. eriodictyol-7-*O*-glucoside; 6. luteolin-7-*O*-glucuronide; 7. cynaroside; 8. luteolin-4'-*O*-glucoside; 9. isorhoifolin; 10. naringenin-7-*O*-glucoside; 11. cosmosiin; 12. rosmarinic acid; 13. salvianolic acid B; 14. luteolin; 15. apigenin.

The comparative analysis of the phenolic compounds in wild and cultivated samples of *D. palmatum* showed that the total content of identified compounds in the cultivated sample was 28% lower (17.571 mg/g *vs*. 24.503 mg/g in wild sample) (Table 2).

It should be noted that the set of compounds detected in the cultivated sample was close to those of the wild sample, except for a significantly higher content of biosides in the cultivated samples (2.735 mg/g). This indicates the reproducibility of *D. palmatum* composition under artificial culture conditions.

Chromatographic study of the aerial parts of *D. palmatum* showed an uneven distribution of the phenolic compounds in the plant. The maximum content of identified compounds was observed in the leaves (37.825 mg/g), minimum in the flowers (11.251 mg/g) and stems contained an intermediate level (20.959 mg/g) (Table 1).

The flavonoids were concentrated in the leaves (35.482 mg/g) and phenylpropanoids accumulated in the stems of plant (3.982 mg/g). The leaves showed a high content of all flavonoidal groups except aglycone and flavonol-biosides, which were concentrated in the flowers (2.843 mg/g) and stems (5.565 mg/g), respectively. The dominating flavonoids in flowers were cynaroside (5.062 mg/g), luteolin (1.998 mg/g) and cosmosiin (1.410 mg/g); in leaves—cynaroside (25.172 mg/g), cosmosiin (7.874 mg/g) and scolymoside (1.240 mg/g); in stems—cynaroside (6.727 mg/g), scolymoside (4.188 mg/g) and cosmosiin (1.640 mg/g). A high content of such rare flavonoids such as isorhoifolin, luteolin-7-*O*-glucuronide, and luteolin-4'-*O*-glucoside were detected in the stems of plants (1.377, 0.580, and 0.376 mg/g, respectively). The organ with maximal phenylpropanoid content was the stem (3.982 mg/g) characterised by the highest concentration of rosmarinic acid (2.791 mg/g, that is 4.1 times more than in the leaves and 9 times higher than in the flowers. Unlike rosmarinic acid, salvianolic acid B accumulates in leaves (1.662 mg/g). The detected concentrations of 3-*O*-caffeoylquinic acid, caffeic acid and cichoric acid were found only in the stems. The distribution of phenolics in the organs of the cultivated sample of *D. palmatum* was similar to that of the wild sample (Table 2). However, for a number of compounds, higher levels were found in cultivated samples. For example, stems of the cultivated sample were enriched with caffeic acid, scolymoside, and eriodictyol-7-*O*-glucoside. In the leaves of wild sample, trace amounts of cichoric acid were observed, whereas in the cultivated plant, the concentration of this compound was up to 0.356 mg/g. The above results demonstrate that *D. palmatum* is a plant with a high content of flavonoids.

**Table 1:** Content of phenolic compounds in wild samples of *D. palmatum* (mg/g of dry plant weight).

Compound	Average amount ± SD			
	Herb	Flowers	Leaves	Stems
3-*O*-Caffeoylquinic acid	0.052 ± 0.001 ^a^	tr.	tr.	0.063 ± 0.001 ^a^
Caffeic acid	0.157 ± 0.002 ^b^	tr.	n.d.	0.512 ± 0.005 ^e^
Cichoric acid	0.237 ± 0.003 ^c^	n.d.	tr.	0.289 ± 0.003 ^cd^
Rosmarinic acid	1.614 ± 0.022 ^i^	0.311 ± 0.003 ^cd^	0.681 ± 0.004 ^f^	2.791 ± 0.021 ^j^
Salvianolic acid B	1.456 ± 0.018 ^hi^	0.431 ± 0.004 ^e^	1.662 ± 0.018 ^i^	0.327 ± 0.003 ^d^
Apigenin	0.315 ± 0.004 ^cd^	0.845 ± 0.010 ^g^	0.370 ± 0.003 ^d^	0.497 ± 0.006 ^e^
Cosmosiin	5.683 ± 0.085 ^k^	1.410 ± 0.014 ^hi^	7.874 ± 0.094 ^l^	1.640 ± 0.018 ^i^
Isorhoifolin	0.240 ± 0.003 ^c^	0.295 ± 0.003 ^c^	0.145 ± 0.002 ^b^	1.377 ± 0.016 ^h^
Luteolin	1.484 ± 0.018 ^hi^	1.998 ± 0.026 ^i^	0.252 ± 0.003 ^c^	1.246 ± 0.014 ^h^
Cynaroside	12.075 ± 0.156 ^m^	5.062 ± 0.065 ^k^	25.172 ± 0.276 ^n^	6.727 ± 0.074 ^k^
Luteolin-7-*O*-glucuronide	0.298 ± 0.004 ^c^	0.212 ± 0.002 ^c^	tr.	0.580 ± 0.006 ^e^
Luteolin-4'-*O*-glucoside	0.254 ± 0.003 ^c^	tr.	tr.	0.376 ± 0.001 ^d^
Scolymoside	0.495 ± 0.007 ^e^	0.445 ± 0.005 ^e^	1.240 ± 0.011 ^h^	4.188 ± 0.046 ^k^
Naringenin-7-*O*-glucoside	0.143 ± 0.003 ^b^	0.163 ± 0.002 ^b^	0.272 ± 0.002 ^c^	0.251 ± 0.003 ^c^
Eriodictyol-7-*O*-glucoside	tr.	0.079 ± 0.001 ^a^	0.157 ± 0.002 ^b^	0.095 ± 0.001 ^a^
Total identified compounds	24.503	11.251	37.825	20.959
Phenylpropanoids	3.516	0.742	2.343	3.982
Flavonoids, including	20.987	10.509	35.482	16.977
flavanones	0.143	0.242	0.429	0.346
flavones	20.844	10.267	35.053	16.631
apigenin derivatives	6.238	2.550	8.389	3.514
luteolin derivatives	14.606	7.717	26.664	13.117
alycones	1.799	2.843	0.622	1.743
monoglycosides	19.420	6.926	33.475	9.669
biosides	0.735	0.740	1.385	5.565

All values correspond to mean values ± standard deviation of three replicates. Values with different letters (a–n) indicate statistically significant differences among groups at *p* < 0.05 by one-way ANOVA. tr.—traces (<limit of quantification); n.d.—not detected (<limit of detection).

**Table 2:** Content of phenolic compounds in cultivated samples of *D. palmatum* (mg/g of dry plant weight).

Compound	Average amount ± SD			
	Herb	Flowers	Leaves	Stems
3-*O*-Caffeoylquinic acid	0.043 ± 0.001 ^a^	tr.	tr.	tr.
Caffeic acid	0.246 ± 0.003 ^c^	tr.	tr.	0.755 ± 0.009 ^f^
Cichoric acid	0.122 ± 0.001 ^b^	tr.	0.356 ± 0.003 ^d^	0.203 ± 0.002 ^c^
Rosmarinic acid	0.943 ± 0.012 ^g^	0.139 ± 0.001 ^b^	0.264 ± 0.003 ^c^	1.943 ± 0.021 ^h^
Salvianolic acid B	0.651 ± 0.008 ^ef^	tr.	1.307 ± 0.010 ^gh^	0.171 ± 0.002 ^bc^
Apigenin	0.157 ± 0.002 ^b^	0.182 ± 0.002 ^bc^	tr.	0.312 ± 0.003 ^d^
Cosmosiin	3.253 ± 0.046 ^i^	0.197 ± 0.002 ^bc^	6.073 ± 0.067 ^j^	1.002 ± 0.012 ^g^
Isorhoifolin	0.273 ± 0.004 ^c^	tr.	0.101 ± 0.001 ^b^	0.340 ± 0.004 ^d^
Luteolin	0.298 ± 0.004 ^c^	0.512 ± 0.004 ^e^	0.396 ± 0.003 ^d^	0.203 ± 0.002 ^c^
Cynaroside	8.263 ± 0.132 ^k^	0.545 ± 0.007 ^e^	15.682 ± 0.173 ^l^	2.972 ± 0.032 ^hi^
Luteolin-7-*O*-glucuronide	0.412 ± 0.005 ^de^	tr.	0.605 ± 0.007 ^ef^	0.143 ± 0.001 ^b^
Luteolin-4′-*O*-glucoside	0.337 ± 0.004 ^d^	tr.	0.568 ± 0.006 ^e^	0.123 ± 0.001 ^b^
Scolymoside	2.462 ± 0.032 ^h^	0.083 ± 0.001 ^ab^	0.749 ± 0.009 ^f^	5.266 ± 0.058 ^j^
Naringenin-7-*O*-glucoside	0.111 ± 0.002 ^b^	0.114 ± 0.001 ^b^	1.204 ± 0.014 ^gh^	0.083 ± 0.001 ^ab^
Eriodictyol-7-*O*-glucoside	tr.	tr.	tr.	0.212 ± 0.002 ^c^
Total identified compounds	17.571	1.772	27.305	13.728
Phenylpropanoids	2.005	0.139	1.927	3.072
Flavonoids, including	15.566	1.633	25.378	10.656
flavanones	0.111	0.114	1.204	0.295
flavones	15.455	1.519	24.174	10.361
apigenin derivatives	3.683	0.379	6.174	1.654
luteolin derivatives	11.772	1.114	18.000	8.707
alycones	0.455	0.694	0.396	0.515
monoglycosides	13.158	0.856	24.132	4.535
biosides	2.735	0.083	0.850	5.606

All values correspond to mean values ± standard deviation of three replicates. Values with different letters (a–l) indicate statistically significant differences among groups at *p* < 0.05 by one-way ANOVA. tr. —traces (<limit of quantification); n.d. —not detected (<limit of detection).

### 2.3. Antioxidant Activity of *D. palmatum*

Experimental investigations of the ethanolic extracts from wild (WSE) and cultivated samples (CSE) of *D. palmatum* herb were conducted using the traditional assays: total antioxidant capacity; 2,2-diphenyl-1-picrylhydrazyl radical (DPPH^•^) scavenging activity; 2,2'-azino-bis(3-ethylbenzthiazoline-6-sulphonic acid) radical (ABTS^•+^) scavenging activity, bromine radical (Br^•^) scavenging activity; carotene bleaching assay; nitric oxide (NO) inactivating activity; hydrogen peroxide (H_2_O_2_) inactivating activity; ferrous (II) ions (Fe^2+^) chelating activity; ferric reducing antioxidant power; erythrocyte membrane stabilising activity (Table 3). All experiments include the determination and comparative estimation of the same antioxidant factors for cynaroside, the predominant component of *D. palmatum* with known antioxidant activity [10].

**Table 3:** Antioxidant activity of ethanolic extracts from wild (WSE) and cultivated samples (CSE) of *D. palmatum* and cynaroside ^a^.

Method ^b^	WSE	CSE	Cynaroside
TAC, mg caffeic acid g^−1^	312.44 ± 6.87 ^i^	284.63 ± 5.69 ^i^	623.16 ± 13.08 ^ii^
DPPH^•^ SA, IC_50_, μg/mL	12.73 ± 0.31 ^iii^	18.62 ± 0.45 ^iii^	17.63 ± 0.38 ^iii^
ABTS^•+^ SA, IC_50_, μg/mL	6.35 ± 0.16 ^iv^	10.78 ± 0.28 ^iv^	9.38 ± 0.23 ^iv^
Br^•^ SA, mg cynaroside g^−1^	389.74 ± 8.18 ^v^	247.86 ± 4.95 ^v^	1000
O_2_^•−^-SA, IC_50_, μg/mL	19.37 ± 0.50 ^vi^	28.63 ± 0.77 ^vi^	14.84 ± 0.41 ^vi^
CBA, IC_50_, μg/mL	1.64 ± 0.05 ^vii^	3.38 ± 0.11 ^vii^	10.28 ± 0.35 ^viii^
NO-IA, IC_50_, μg/mL	29.33 ± 1.20 ^ix^	41.77 ± 1.67 ^ix^	>100
H_2_O_2_-IA, mM g^−1^	2.03 ± 0.09 ^x^	1.18 ± 0.05 ^x^	0.52 ± 0.03 ^x^
Fe-CA, IC_50_, μg/mL	30.91 ± 1.08 ^xi^	48.11 ± 1.62 ^xi^	>100
FRAP, mM Fe^2+^ g^−1^	22.25 ± 0.85 ^xii^	12.22 ± 0.48 ^xiii^	9.53 ± 0.47 ^xiii^
EM-SA, IC_50_, μg/mL	14.07 ± 0.70 ^xiv^	51.60 ± 2.68 ^xv^	25.67 ± 1.23 ^xiv^

^a^ Average of three analyses (±SD); ^b^ TAC—total antioxidant capacity; DPPH^•^ SA—DPPH^•^ radical scavenging activity; ABTS^•+^ SA—ABTS^•+^ radical scavenging activity; Br^•^ SA—Br^•^ radical scavenging activity; O_2_^•−^ SA—superoxide anion radical scavenging activity; CBA—carotene bleaching assay; NO-IA—NO inactivating activity; H_2_O_2_-IA—H_2_O_2_ inactivating activity; Fe-CA—Fe^2+^ chelating activity; FRAP—ferric reducing antioxidant power; EM-SA—erythrocyte membrane stabilising activity. All values correspond to mean values ± standard deviation of three replicates. Values with different letters (i–xiv) indicate statistically significant differences among groups at *p* < 0.05 by one-way ANOVA.

The values of total antioxidant capacity of WSE and CSE were 312.44 and 284.63 mg caffeic acid per gram of extract, respectively. These values indicate a high antioxidant potential of the studied extracts. The radical scavenging activity of WSE and CSE against radicals of different nature (organic, inorganic, neutral, and charged) was much expressed, and in some cases was higher than the same parameter of cynaroside (DPPH^•^ and ABTS^•+^ scavenging activities). This data classifies *D. palmatum* extracts as a radical scavenger. The examination of the influence of WSE and CSE on the oxidative destruction of β-carotene in the oleic acid-DMSO-H_2_O_2_ system demonstrated a high value of antioxidant activity, with IC_50_ = 1.64 and 3.38 μg/mL, respectively. The feature of this system is the ability to investigate the influence of sample on the presence of a complex of damaging factors, including H_2_O_2_, O_2_^•−^, OH^•−^, and alkyl-radicals that form in this *in vitro* system. The efficiency of cynaroside in this assay was slightly lower (10.28 μg/mL). The activity of WSE in the NO and H_2_O_2_ inactivating assays and both the extracts in Fe^2+^ chelating activity and FRAP assays were characterised as very high because the activity of cynaroside was quite low. The final stage of the biological study was to investigate the ability of the extracts to protect living cells (RBCs) from oxidative damage caused by the influence of Fenton's reagent. The presence of Fenton's reagent causes a cascade of negative reactions leading to loss of integrity of erythrocyte membranes, which in turn lead to cellular death [11]. The WSE has successfully demonstrated high protective properties (IC_50_ = 14.07 μg/mL) exceeding those of the reference substance (IC_50_ = 25.67 μg/mL).

The obtained chemical information about *D. palmatum* herb allows characterizing the isolated phenolic compounds as responsible factors stipulated the antioxidant properties of the total extract. Earlier, the expressed antioxidant activity of the aglycone and glucosides of luteolin and apigenin [12], as well as derivatives of caffeic acid [13], have been shown by various researchers. To confirm the leading role of flavonoids and phenylpropanoids found in *D. palmatum* herb in formation of the antioxidant effect of the total extracts, we applied the original methodical approach. The identification of antioxidants present in the extracts realized after HPLC-separation of the extracts samples pretreated with excess of DPPH^•^ or ABTS^•+^ radicals (DPPH-HPLC or ABTS-HPLC). The reaction between an antioxidant and a radical results in the oxidation of the antioxidant, that leads to a decrease of the corresponding peak areas in the chromatograms. Comparison of the HPLC chromatograms of untreated and radical-treated samples allows to determine the most active compounds. Previously, some plant species, including *Lonicera japonica* Thunb. [14], *Selaginella sinensis* (Desv.) Spring. [15], *Artemisia gmelinii* Webb. ex Stechm. [16], *Pueraria lobata* (Wild.) Ohwi [17], *Arachis hypogaea* L. [18] and *Eucommia ulmoides* Oliv. [19], were successively investigated by this method.

**Figure 4:**
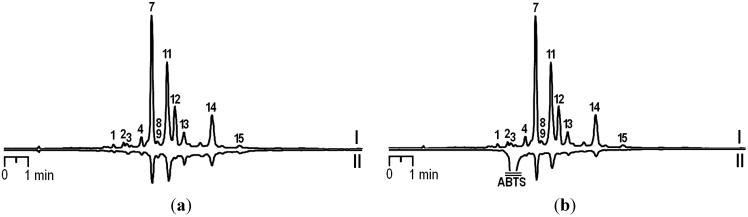
HPLC chromatograms of ethanolic extracts from wild samples of *D. palmatum* at 270 nm before (I) and after (II) prechromatographic reaction with DPPH^•^ (**a**) and ABTS^•+^ radicals (**b**).

Chromatograms of ethanolic extract from *D. palmatum* herb (wild sample) spiking with DPPH^•^ and ABTS^•+^ radicals are shown in Figure 4a,b, respectively, which present obviously reduced peak areas for some compounds in comparison with untreated sample. Therefore, seven compounds, caffeic acid (peak 2), scolymoside (peak 4), cynaroside (peak 7), cosmosiin (peak 11), rosmarinic acid (peak 12), salvianolic acid B (peak 13) and luteolin (peak 14), in extract of *D. palmatum* herb possessed antioxidant activity. It should be noted that the peaks of luteolin, cynaroside, cosmosiin, rosmarinic acid and salvianolic acid B decreased more sharply than the other peaks, so it may be concluded that they are the major active compounds.

1. 3-*O*-Caffeoylquinic acid; 2. caffeic acid; 3. cichoric acid; 4. scolymoside; 7. cynaroside; 8. luteolin-4'-*O*-glucoside; 9. isorhoifolin; 11. cosmosiin; 12. rosmarinic acid; 13. salvianolic acid B; 14. luteolin; 15. apigenin.

The results of the examination of the antioxidant activity of *D. palmatum* herb indicate that the extracts from this plant strongly scavenge free radicals (DPPH^•^, ABTS^•+^, Br^•^, O_2_^•−^) and NO molecules, inactivate hydrogen peroxide, and chelate Fe^2+^ ions *i.e.*, exhibit a high antioxidant potential in processes involving hydrogen atom transfer reactions, electron-transfer reaction and other mechanisms. Comparative analysis of the data indicates that in some cases, the antioxidant activity of the extracts was similar to or exceeded the activity of reference antioxidant, cynaroside.

The unique feature of this plant species is its ability to simultaneously accumulate active compounds such as cynaroside and cosmosiin, which are known to be compounds with high biological activity. In particular, cosmosiin possesses anti-inflammatory [20], insulin-mimetic [21], and cancer prevention activity [22]; cynaroside possesses anti-atherosclerotic [23], anti-inflammatory [24], anti-diabetic [25], and cardioprotective activity [26]. Recently, the effects of cosmosiin against liver injury caused by CCl_4_ were investigated [27]. It was shown that the application of this phytocomponent not only suppressed the elevation of hepatic stress-indicators, glutamic pyruvic transaminase, glutamic oxaloacetic transaminase, malonic dialdehyde and hydroxydeoxyguanosine, and inhibited the decrease of the reduced glutathione level, but also reduced hepatocyte damage. In addition, in a model of hepatic oxidative injury the normalizing action of cosmosiin on the concentrations of aspartate transaminase, alanine transaminase, alkaline phosphatase, glutamate, total bilirubin, lactate dehydrogenase and total serum protein was demonstrated [28]. The results obtained thus suggest that cosmosiin has protective effects against chemical-induced hepatic damages. Cynaroside is the strongest bioactive component responsible for the detoxification of bromobenzene-induced hepatic lipid peroxidation [29]. A significant protective effect of cynaroside reflects in its reduction of lipid peroxide levels and enhanced activity of epoxide hydrolase, a toxicant agent-removing enzyme, rather than by acting on the epoxide-producing system. The modern data of pharmacological activity of cosmosiin and cynaroside are in good agreement with the ethnoscientifical information concerning the use of *D. palmatum* as a hepatoprotective agent.

## 3. Experimental

### 3.1. General

Elemental composition was determined using MAT 8200 spectrometer (Thermo Finnigan, Waltham, MA, USA). UV spectra were recorded using a SF-2000 spectrophotometer (OKB Specter, St. Petersburg, Russia). Optical rotations were measured on 341 Series polarimeter (Perkin Elmer, Waltham, MA, USA). MS spectra were registered on a LCQ mass spectrometer (Thermo Finnigan). NMR spectra were recorded on a VXR 500S spectrometer (Varian, Palo Alto, CA, USA). Culometric analysis was performed on an Expert-006 culometric titrator (Econix-Expert, Moscow, Russia). Column chromatography was performed over silica gel 60 (NP-SiO_2_; 230–400 mesh, Merck, Whitehouse Station, NJ, USA), Sephadex LH-20 (25–100 μm, Pharmacia, Uppsala, Sweden), polyamide Woelm (Waters Associates, Inc., Framingham, MA, USA), octadecyl-functionalised silica gel (RP-SiO_2_; Sigma-Aldrich, St. Louis, MO, USA), and Amberlite XAD7HP (Sigma-Aldrich, St. Louis, MO, USA). Finally, pTLC was performed on Sorbfil-A silica gel TLC plates (layer thickness 2 mm; Imid Ltd., Krasnodar, Russia). All chemicals were analytical-grade.

### 3.2. Plant Material

The wild samples of *D. palmatum* were collected in the vicinity of Tomtor village in Oĭmyakon District, Sakha Republic of the Russian Federation (63°26'34''N, 143°10'24''E; on 20 July 2012; voucher specimen No Lm/h-805/0415). The cultivated plants of *D. palmatum* were grown from authenticated seeds obtained from Tsitsin’s Botanical Garden, Russian Academy of Science (Moscow, Russia) by cultivation in the fields of the Botanical Garden of Institute of Biology (IB, Yakutsk, Russia; 62°08'60''N, 129°61'67''E). The aboveground parts of the plants were collected in the middle of July 2012, then dried and stored at 4 °C in the IB Plant Repository (voucher specimen No Lm/h-514/0086). For analytical HPLC total probes of the flowers, leaves, stems and herb from 15 specimens of *D. palmatum* were used.

### 3.3. Extraction and Isolation

The wild samples of *D. palmatum* (0.85 kg) were air-dried, ground, and extracted with 60% EtOH (8.5 L) at 80 °C three times and the extracts were concentrated under reduced pressure to yield 272 g of crude extract. The crude extract was resuspended in water (1:5, v/v) and successively partitioned with hexane, CHCl_3_, EtOAc, and *n*-BuOH. The organic layers were dried *in vacuo* to yield 59.5, 25.5, 42.5 and 102.4 g of hexane (F1), CHCl_3_ (F2), EtOAc (F3) and *n*-BuOH (F4) fraction residues respectively. The F2 fraction (20 g) was chromatographed over silica column (3 × 120 cm), eluting with CHCl_3_-MeOH (100:0→0:100) to obtain 10 fractions (frs. F2/1–F2/10). Fractions F2/3–F2/4 were combined and chromatographed on a silica column (1.5 × 70 cm), eluting with hexane-EtAc (100:0→70:30) to obtain 10 fractions (frs. F2/3–4/1 to F2/3–4/10). Frs. F2/3–4/2 to F2/3–4/4 were combined and crystallized from hexane-CHCl_3_ to give ursolic acid (**22**; 6.73 g) [30]; frs. F2/3–4/5 to F2/3–4/6 after crystallisation from CHCl_3_ yielded oleanolic acid (**23**; 108 mg) [31]. Frs. F2/6–F2/8 were combined and chromatographed on a Sephadex LH-20 column (2 × 100 cm), eluting with CHCl_3_-MeOH (100:0→0:100) to obtain 10 fractions (frs. F2/6–8/1 to F2/6–8/10). Frs. F2/6–8/3 to F2/6–8/5 were combined and separated using pTLC (solvent: toluene-EtOAc-HCOOH 6:3:1) to give umbelliferon (**7**; 24 mg) and aesculetin (**8**; 32 mg) [31]. Frs. F2/6–8/6 to F2/6–8/7 were combined and crystallized from MeOH to give apigenin (**9**; 86 mg) [32]. Fr. F3 (41 g) was subjected on a XAD7HP column (600 g), eluting with H_2_O (10 L), 40% EtOH (14 L) and 90% EtOH (8 L). These elutes were brought dried *in vacuo* to yield 2.6, 29.4 and 8.1 g of H_2_O (F3-1), 40% EtOH (F3-2) and 90% EtOH fraction (F3-3) residue respectively. Fr. F3-2 was chromatographed on polyamide column (4 × 120 cm), eluting with H_2_O-MeOH (100:0→0:100) to obtain 10 fractions (fr. F3-2/1 to fr. F3-2/10). Frs. F3-2/2 and F3-2/3 were separated on Sephadex LH-20 column (3 × 110 cm), eluting with MeOH-H_2_O (100:0→0:100) to obtain caftaric acid (**5**; 27 mg) [33], 3-*O*-caffeoylquinic acid (**2**; 85 mg) and caffeic acid (**1**; 64 mg) [34], cichoric acid (**6**; 39 mg) [33], respectively. Frs. F3-2/4–F3-2/6 were combined and separated on RP-SiO_2_ column (3 × 100 cm), eluting with H_2_O-MeCN (100:0→0:100) to give apigenin-7-*O-*β-d-glucuronopyranoside (**11**; 18 mg), cosmosiin (**10**; 242 mg), cynaroside (**14**; 1.84 g), luteolin-7-*O-*β-d-glucuronopyranoside (**15**; 37 mg), luteolin-4'-*O-*β-d-glucopyranoside (**16**; 27 mg) [32]. Frs. F3-2/7–F3-2/9 were combined and separated on RP-SiO_2_ column (3 × 100 cm), eluting with H_2_O-MeCN (100:0→0:100) to give naringenin-7-*O-*β-d-glucopyranoside (**19**; 31 mg) and eriodictyol-7-*O-*β-d-glucopyranoside (**21**; 24 mg) [32]. Fr. F3-3 was chromatographed on silica column (2.5 × 90 cm), eluting with EtAc-MeOH (100:0→70:30) to obtain luteolin (**13**; 104 mg), naringenin (**18**; 11 mg) and eriodictyol (**20**; 9 mg) [32]. Fr. F4 (80 g) was subjected on a XAD7HP column (900 g), eluting with H_2_O (25 L), 40% EtOH (30 L) and 90% EtOH (6 L). The elutes were dried *in vacuo* to yield 42.7, 25.4 and 10.1 g of H_2_O (F4-1), 40% EtOH (F4-2) and 90% EtOH (F4-3) fraction residues respectively. Fr. F4-1 is characterised by trace amount of phenolic compounds and was not investigated. Fr. F4-2 was crystallised from 50% EtOH to give yellow precipitate (11.4 g) which was chromatographed on polyamide column (3 × 80 cm), eluting with H_2_O-MeOH (100:0→0:100) to obtain 10 fractions (fr. F4-2/1–fr. F4-2/10). Frs. F4-2/2–F4-2/3 were combined and crystallised from 50% EtOH to give scolymoside (**17**; 92 mg) [35] and frs. F4-2/5–F4-2/6 after the same procedure give isorhoifolin (**12**; 34 mg) [32]. Fr. F4-3 was chromatographed on pHPLC (Summit HPLC-system with UV-V is detector (Dionex, Sunnyvale, CA, USA), column LiChrosorb RP-18 (4.6 × 250 mm, 5 μm, Merck), T 35 °C, flow rate 1 mL/min; solvent, linear gradient of 5%–80% of MeCN in H_2_O for 90 min; detector at 330 nm) to give rosmarinic acid (**3**; 108 mg) and salvianolic acid B (**4**; 28 mg) [36].

### 3.4. Microcolumn HPLC-UV

The dried and powdered plant samples (200 mg) were extracted with 60% ethanol (5 mL) in an ultrasonic bath for 40 min at 45 °C. The extracted solutions were filtered through a 0.22 μm PTFE syringe filter before injection into the HPLC system for analysis. HPLC analysis was performed on a MiLiChrom A-02 microcolumn chromatograph (Econova, Novosibirsk, Russia) coupled with UV-detector, using a ProntoSIL-120-5-C18 AQ column (2 × 75 mm, ∅ 5 μm; Metrohm AG, Herisau, Switzerland). Mobile phase A was 0.2 M LiClO_4_ in 0.006 M HClO_4_ and mobile phase B was acetonitrile. The injection volume was 1 μL, and elution at 150 μL/min with gradient programme (0–7.5 min, 11%–18% B; 7.5–13.5 min, 18% B; 13.5–15 min, 18%–20% B, 15–18 min, 20%–25% B; 18–24 min, 25% B; 24–30 min, 25%–100% B). Detector wavelength was 270 nm. Reference compounds with purity greater than 96% were used. This included 3-*O*-caffeoylquinic acid, caffeic acid, cichoric acid, rosmarinic acid, salvianolic acid B, apigenin, luteolin from Sigma-Aldrich; cosmosiin, isorhoifolin, cynaroside, luteolin-4'-*O*-glucoside, naringenin-7-*O*-glucoside, eriodictyol-7-*O*-glucoside from Extrasynthese (Lyon, France); luteolin-7-*O*-glucuronide from ChemFaces (Wuhan, China); scolymoside was isolated previously from *Lophanthus chinensis* Benth. herb [35].

### 3.5. Preparation of the Extracts WSE and CSE

The total herb of wild or cultivated samples of *D. palmatum* were air-dried and powdered in a mechanical grinder. The powdered total herb was weighted accurately (100 g), extracted twice with 60% ethanol (1.5 L) in an ultrasonic bath for 90 min at 45 °C. The extracted solutions were filtered through cellulose filter and evaporated *in vacuo* until dryness using rotary evaporator. The extracts yields are 35.63% (w/w; from wild sample, WSE) and 29.15% (w/w; from cultivated sample, CSE).

### 3.6. Antioxidant Activity Assays

The total antioxidant capacity (TAC) was determined using the phosphomolybdic acid method [37]; the DPPH^•^ radical scavenging activity (DPPH-SA) was assessed as described by Asker and Shawky [38]; the ABTS^•+^ radical scavenging activity (ABTS^•+^-SA) was measured using the method of Ding *et al*. [39]; the Br^•^ radical scavenging activity (Br^•^-SA) was determined using culometric method of Abdulin *et al*. [40] with electrogenerated bromine radicals; the determination of superoxide anion scavenging activity (O_2_^•−^-SA) was measured in phenazine methosulphate-nicotinamide adenine dinucleotide-nitroblue tetrazolium systems using the method of Ozen *et al*. [41]; β-carotene bleaching assay (CBA) was performed in β-carotene-oleic acid-DMSO-H_2_O_2_-system [42]; the NO inactivating activity (NO-IA) was measured using the sodium nitroprusside method [43]; the H_2_O_2_ inactivating activity (H_2_O_2_-IA) was measured using the method of Badami and Channabasavaraj [44]; the chelating activity for Fe^2+^ ions (Fe-CA) was measured by the *o*-phenanthroline method [45]; the ferric reducing antioxidant power (FRAP) was determined using 2,4,6-tripyridyl-s-triazine (TPTZ) method [46]; the erythrocyte membrane stabilising activity (EM-SA) was determined using Fenton-induced hemolytic method [47].

### 3.7. DPPH-HPLC-UV (ABTS-HPLC-UV) Procedure

Briefly, *D. palmatum* extract solution in 60% ethanol (500 μL, 30 mg/mL) were added to DPPH^•^ radical solution in methanol (500 μL, 20 mg/mL) or ABTS^•+^ radical solution in phosphate buffer (pH 7.4; 5 mg/mL). The mixture was shaken for few seconds and left to stand in the dark for 30 min at room temperature. Then the sample was filtered through a 0.22 μm membrane filter. The untreated sample was prepared by adding *D. palmatum* extract solution in 60% ethanol (500 μL, 30 mg/mL) to methanol or phosphate buffer (pH 7.4) (500 μL). HPLC analysis was performed on a MiLiChrom A-02 (Econova) microcolumn chromatograph coupled with a UV-detector, using a ProntoSIL-120-5-C18 AQ column (2 × 75 mm, ∅ 5 μm; Metrohm AG). Mobile phase A was 0.2 M LiClO_4_ in 0.006 M HClO_4_ and mobile phase B was acetonitrile. The injection volume was 1 μL, and elution at 150 μL/min with gradient programme (0–15 min, 10%–80% B). Detector wavelength was 270 nm.

### 3.8. Statistical Analysis

Statistical analyses were performed using a one-way analysis of variance (ANOVA), and the significance of the mean difference was determined by Duncan’s multiple range test. Differences at *p* < 0.05 were considered statistically significant. The results were presented as mean values ± SD (standard deviations) of the three replicates.

## 4. Conclusions

*Dracocephalum palmatum*, a medicinal plant used by the North-Yakutian nomads, was investigated chemically for the first time. The presence of phenylpropanoids, coumarins, flavonoids and triterpenes in *D. palmatum* herb was shown. A comparative study of wild and cultivated samples demonstrated the lower level of the phenolic compounds in cultivated plants, possibly due to the fact that extreme environmental conditions increased the amount of these active compounds. However, both wild and cultivated plants could be used. The organ-specific distribution of phenolic compounds and the finding of eight new compounds specific to *D. palmatum*, which are not found in other species of *Dracocephalum*, suggest that this herb is one of the best sources of these phenolic compounds for humans. The high level of the antioxidant activity of the crude extract and isolated compounds was revealed using different *in vitro* methods. Obtained results confirmed the ethnopharmacological use of *D. palmatum* as a natural phytotherapeutic agent.
